# Pyrosequencing Reveals Fungal Communities in the Rhizosphere of Xinjiang Jujube

**DOI:** 10.1155/2015/972481

**Published:** 2015-01-05

**Authors:** Peng Liu, Xiao-Hui Wang, Jian-Gui Li, Wei Qin, Cheng-Ze Xiao, Xu Zhao, Hong-Xia Jiang, Jun-Kang Sui, Rong-Bo Sa, Wei-Yan Wang, Xun-Li Liu

**Affiliations:** ^1^College of Forest, Shandong Agricultural University, No. 61, Daizong Street, Taian, Shandong 271018, China; ^2^College of Life Science, Shandong Agricultural University, Taian, Shandong 271018, China; ^3^Research Institute of Forest in Xinjiang Agricultural University, Urumqi, Xinjiang 830052, China

## Abstract

Fungi are important soil components as both decomposers and plant symbionts and play a major role in ecological and biogeochemical processes. However, little is known about the richness and structure of fungal communities. DNA sequencing technologies allow for the direct estimation of microbial community diversity, avoiding culture-based biases. We therefore used 454 pyrosequencing to investigate the fungal communities in the rhizosphere of Xinjiang jujube. We obtained no less than 40,488 internal transcribed spacer (ITS) rDNA reads, the number of each sample was 6943, 6647, 6584, 6550, 6860, and 6904, and we used bioinformatics and multivariate statistics to analyze the results. The index of diversity showed greater richness in the rhizosphere fungal community of a 3-year-old jujube than in that of an 8-year-old jujube. Most operational taxonomic units belonged to Ascomycota, and taxonomic analyses identified Hypocreales as the dominant fungal order. Our results demonstrated that the fungal orders are present in different proportions in different sampling areas. Redundancy analysis (RDA) revealed a significant correlation between soil properties and the abundance of fungal phyla. Our results indicated lower fungal diversity in the rhizosphere of Xinjiang jujube than that reported in other studies, and we hope our findings provide a reference for future research.

## 1. Introduction

Fungi are important in soils as decomposers and plant symbionts [1]; it is thus important to understand the composition of their complex communities [2]. They play essential roles in many aspects of ecosystem development, function, and stability [3]. Understanding fungal diversity, community structure, and spatial patterns is one of the central issues in soil microbial ecology [4]. Microbial diversity is measured by a variety of techniques, including traditional plate culture, phospholipid and fatty acid analysis, phyloarrays, fingerprinting techniques, other DNA-based techniques such as restriction analysis and Sanger sequencing, and denaturing gradient gel electrophoresis of microbial proteins [5, 6]. Plate culture is the primary method used to study microbial communities, but because of the methodological limitations associated with it [7, 8], only few studies have addressed the diversity of microbial communities. Although the vast majority of microbial soil species is unculturable, the development of culture-independent methods has yielded important insights into the phylogenetic diversity of soil microorganisms [9]. High-throughput sequencing technologies now play a significant role in microbial ecology studies [10] and next-generation techniques such as 454 pyrosequencing facilitate the detection of unculturable microbial communities [11, 12].

Pyrosequencing is faster, approximately 20–30 times less expensive than Sanger sequencing, and does not require cloning [13]. The 454 pyrosequencing technique has recently been used to characterize fungal diversity. Buée et al. [14] used this technique for forest soils to reveal unexpectedly high fungal diversity; Lim et al. [13] assessed soil fungal communities by using pyrosequencing, as did Jumpponen and Jones [15] to characterize the fungal communities in* Quercus *spp. Ectomycorrhizas and revealed seasonal dynamics in urban and rural sites. The 454 pyrosequencing platform is a new type of second-generation sequencing technology; it produces 25 million base reads in a single run with an accuracy of 99% [16]. It is rapid, flexible, and inexpensive, and it does not require a cloning step [17]. This technology permits metagenomic analyses that exceed the capacity of traditional Sanger sequencing-based approaches by several orders of magnitude and offers the possibility of detecting very rare phylotypes [18].

The Xinjiang Uygur Autonomous Region, which is the largest province in China, is situated in the hinterland of Asia and is characterized by very low precipitation and high evaporation [19]. These unique climatic characteristics of Xinjiang, such as little rainfall with periodic drought, abundant sunshine, and substantial differences between day and night temperatures [20], create conditions in which the composition and distribution of soil fungal communities may vary significantly. For example, Feng-gang et al. [21] analyzed the culturable fungal diversity in the rhizosphere of healthy and diseased cotton in southern Xinjiang. Chinese jujube is a common traditional Chinese medicine and food that has been used for thousands of years [22]. Jujube is one of the main economic crops in Xinjiang, as the quality of Xinjiang jujube is better than that in other regions because of the unique climatic conditions. However, the fungal communities in the rhizosphere of Xinjiang jujube have never been studied previously.

In this study, we used 454 pyrosequencing to analyze the fungal communities in the jujube rhizosphere in the three areas of Xinjiang, namely, the prefectures of Hetian, Kashi, and Aksu in China. The objectives of this study were to characterize fungal diversity and community composition and to compare fungal communities from different areas and growth years. We also isolated several fungal jujube pathogens. Finally, the relationship between fungal phyla and soil conditions was tested by redundancy analysis (RDA).

## 2. Materials and Methods

### 2.1. Sample Collection and DNA Extraction

**Figure 1:**
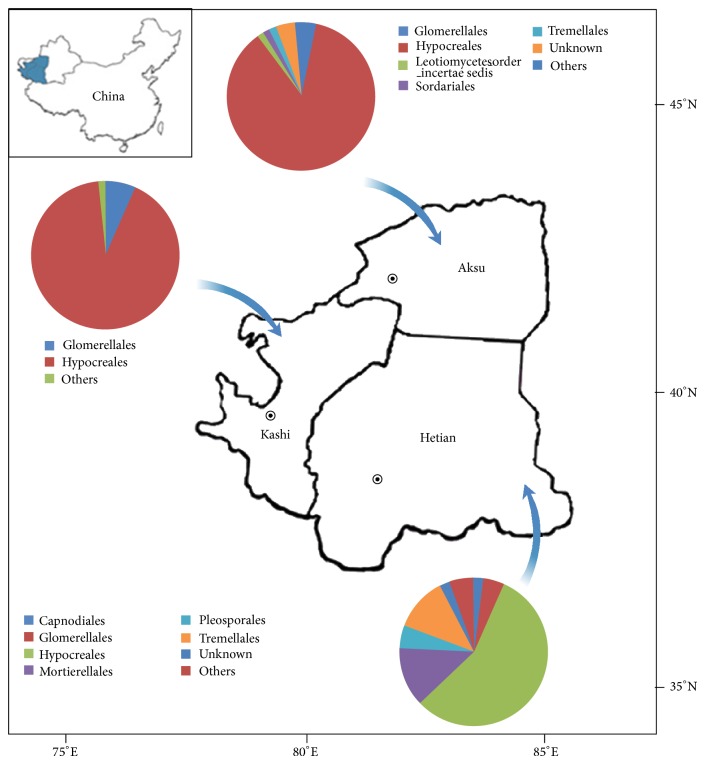
Location of sampling sites of soil samples across the Xinjiang Uygur Autonomous Region of China and relative abundance of the dominant fungal order in 6 soil samples.

Soil samples were collected from the rhizosphere of jujube (variety Junzao) across the southern Xinjiang Uygur Autonomous Region of China (Figure 1). Eighteen samples were collected from the prefectures of Kashi (Ka), Hetian (He), and Aksu (Ak). Samples were identified as He-3, He-8, Ka-3, Ka-8, Ak-3, and Ak-8, where the numbers 3 and 8 represent the rhizosphere of 3- and 8-year-old jujube plants. The three areas from which soil samples were collected were separated by distances of about 460–770 km. Jujube trees aged 3 and 8 years were selected at each site, and three soil samples for each age were collected by five-point sampling. All soil samples were placed in separate sterile plastic bags that were immediately placed on ice and then transported to the laboratory where they were stored at −20°C until DNA extraction. The physicochemical parameters of the soil were measured as follows. Soil moisture content was estimated by the oven dry-weight method [23] and pH was determined using a glass electrode meter in a suspension of 1 g of soil in 5 mL of distilled water. Available phosphorous (P) was extracted using sodium bicarbonate and then measured by the molybdenum blue method. Available potassium (K) was determined by flame photometry [24]. Available nitrogen (N) was determined by potassium persulfate oxidation [25]. Organic matter content was determined as described by Walkey and Black [26]. Total genomic DNA was extracted from soil samples (0.4 g) by using the Soil DNA kit (OMEGA, Bio-Tek, USA), according to the manufacturer's instructions. The DNA extracted from three successive rounds of extraction from each sample was pooled prior to PCR.

### 2.2. PCR Amplification and 454 Pyrosequencing

PCR amplification of the fungal internal transcribed spacer (ITS) rDNA genes from genomic DNA was performed with barcoded primers. The bacterial forward primer was ITS1F (CTTGGTCATTTAGAGGAAGTAA), and the reverse primer was ITS2 (GCTGCGTTCTTCATCGATGC). PCR was performed in a 20 *μ*L reaction mixture containing 1 *μ*L of DNA template (10 ng/*μ*L), 0.4 *μ*L of each primer (10 pmol), 0.15 *μ*L of Ex Taq (TaKaRa), 2 *μ*L of 10x buffer, 1.6 *μ*L of dNTPs (2.5 mM) (TaKaRa), and 13.5 *μ*L of ddH_2_O. Cycling conditions were as follows: initial denaturation at 98°C for 2 min, followed by 35 cycles of 98°C for 15 s, 56°C for 30 s, and 72°C for 40 s, with a final extension at 72°C for 10 min. PCR products were purified using the TaKaRa Agarose Gel DNA Purification kit and quantified with a NanoDrop. A mixture of purified ITS rDNA amplicons from each soil sample was subjected to pyrosequencing on the 454 GS FLX titanium platform (Roche, Basel, Switzerland) at the National Human Genome Centre of China (Shanghai).

### 2.3. Processing of Pyrosequencing Data

Pyrosequencing data were processed using Mothur (version 1.25.1) following the Schloss standard operating procedure [27]. Pyrosequencing reads with ambiguous nucleotides, sequences <200 bp in length, one or more primer mismatches, or two or more barcode mismatches were removed and excluded from further analysis. The filtered sequences were then assigned to soil samples according to the corresponding barcodes with a Bespoke Java program. Operational taxonomic units (OTUs) at the 3% dissimilarity level were determined by comparing the sequences to those in the Silva database (http://www.arb-silva.de/) using Mothur, and the most abundant sequence in each OTU was selected as the representative. Representative sequences were taxonomically classified using a Ribosomal Database Project naive Bayesian rRNA classifier 2.2 [28] with a confidence threshold of 0.8. The relative proportion of a given phylogenetic group with respect to the entire fungal communitywas defined as the number of sequences affiliated with that group divided by the total number of sequences per sample [29].

### 2.4. Bioinformatics and Statistical Analysis

An OTU-based analysis was performed to calculate species richness, diversity, and coverage of each rhizosphere sample, with a cutoff of 3% dissimilarity [29]. The R software (version 2.14.2; R Development Core Team 2009) was used to calculate the Shannon-Wiener index, Simpson's Diversity index, and Evenness index of each sample [30]. Rarefaction curves generated by Mothur were used to compare relative levels of bacterial OTU diversity across all soil samples [29]. The coefficient of community structure or distance coefficient was calculated and used for UPGMA cluster analysis. Venn diagrams were generated using custom Perl scripts [31]. The relationship between bacterial phyla and soil conditions was analyzed by redundancy analysis using R software. All chemical data are expressed as the mean value ± SE. Analysis of variance was performed using SAS 8.0 to test for sample differences in *α*-diversity and soil properties.

## 3. Results

### 3.1. Estimators for Diversity and Species Richness of Fungal Communities

**Figure 2:**
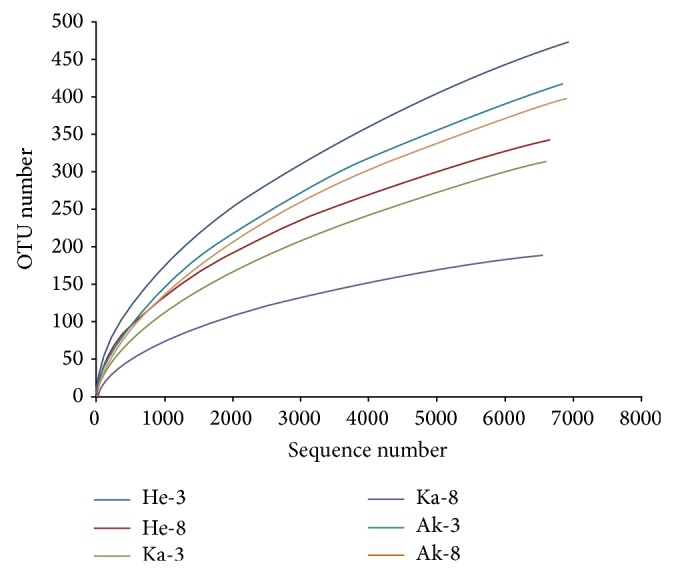
Rarefaction curves of fungal depicting the effect of 3% dissimilarity on the number of OTUs identified in the 6 soil samples.

**Figure 3:**
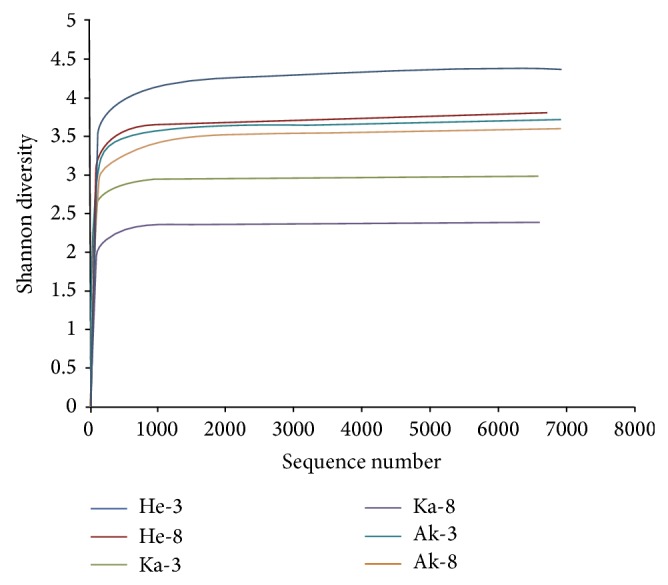
Shannon curves of fungal depicting the effect of 3% dissimilarity on the number of OTUs identified in the 6 soil samples.

For the six sequencing samples, exclusion of low-quality and short sequence reads yielded 40,488 fungal ITS pyrotag reads. Each sample averaged 6,748 reads; the number of each sample was 6943, 6647, 6584, 6550, 6860, and 6904. Using a 3% dissimilarity cut-off for clustering, thereads were grouped into different OTUs (Table 1). The number of fungal OTUs was generally higher in soil samples collected from the rhizosphere of 3-year-old jujube from the same area, with the greatest number of OTUs (474) in sample He-3and the least (189) in sample Ka-8. Rarefaction curves for the fungal community at distance levels of 0.03 had not reached an asymptote (Figure 2). Thus, the sequencing capability did not fully represent the number of different fungal communities, but by combining the rarefaction curves with the Shannon diversity index, we found that, with the increase in sequencing number, the Shannon diversity curves (Figure 3) approached a plateau. Therefore, the data were sufficient to allow an analysis of fungal communities. The indices of diversity and richness of fungi in soil samples are shown in Table 1. The ACE and Chao values, which are indicators of species richness, were greater for the rhizosphere fungal community of the 3-year-old jujube than those for the 8-year-old jujube rhizosphere. Shannon diversity index values showed a similar trend in fungal diversity.

### 3.2. Fungal Community Composition and Structure Analysis

**Figure 4:**
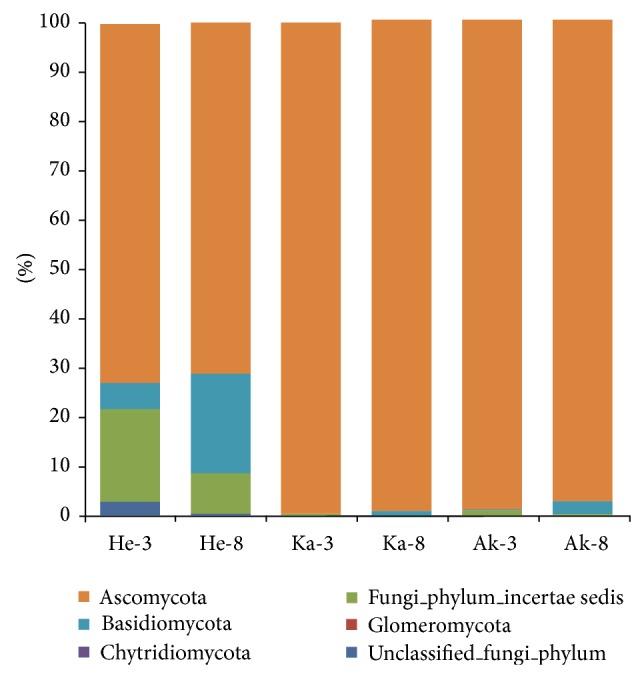
Relative abundance of the dominant fungal phyla in 6 soil samples.

All fungal sequences were classified at the phylum level down to the genus level according to the Mothur program. Of the classifiable sequences, four phyla were identified (Figure 4), with* Ascomycota *representing the most dominant phyla and accounting for 73.2%, 71.7%, 98.9%, 97.8%, 97.6%, and 97.1% of the six soil samples. The distribution of Basidiomycota varied, accounting for 20.3% in sample He-8, but only 0.058% in Ak-3. Some phyla existed in only specific soil samples. Chytridiomycota was found only in samples He-3 and Ak-3. Glomeromycota was present only in He-3 and constituted 1 OTU. In addition, the proportion of unclassified fungi was relatively large in He-3 (21.7%) and He-8 (8.6%).

Down to the order level, the most abundant fungal order was Hypocreales, accounting for 56.3%, 91.9%, and 85.1% of the three sampling areas. The diversity of fungal order was higher in Hetian than in other areas. The Mortierellales and Tremellales were the next most common orders, at 13.1% and 11.6%. The Hetian area also included Glomerellales (4.6%), Pleosporales (4.9%), Capnodiales (2.0%), and unknown (2.2%). In the Kashi area, Hypocreales accounted for 91.9%, Glomerellales was 6.6%, and others were 1.5%; there were no other fungal orders, making the diversity in this area the lowest of the tested regions. Hypocreales(85.1%), Tremellales (1.3%), and Glomerellales (3.2%) were found in Aksu and Hetian; Leotiomycetes (1.3%) was found only in Aksu. Mortierellales was absent from Aksu. We also identified jujube pathogens from the fungal sequence reads and found that the fewest pathogens were in the Xinjiang soil samples (*Aspergillus* and* Schizophyllum*)*. Aspergillus *was found inHetian and Aksu, only in 3-year-old jujube trees. The proportions of* Aspergillus *in He-3 and Ak-3 were 0.1% and 0.4%, respectively.

### 3.3. Comparison of Fungal Communities between Different Sampling Areas

**Figure 5:**
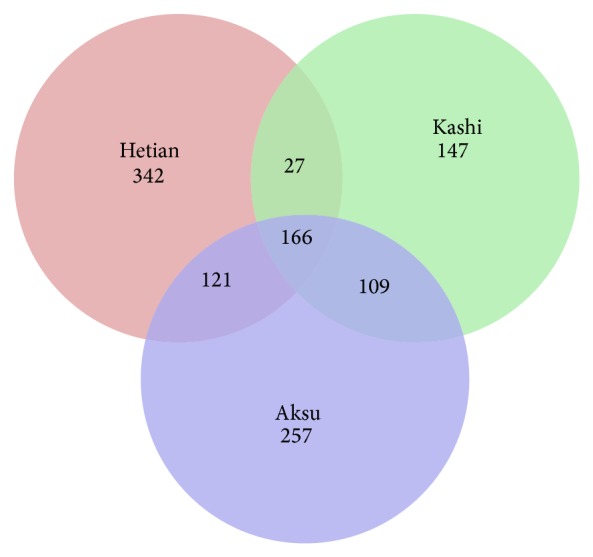
Venn diagram representing the number of fungal OTUs that are unique and shared between the samples from 3 different sampling areas.

Fungal OTUs common to the three sampling areas were represented using a Venn diagram to compare the relationships between the three communities (Figure 5). The number of fungal OTUs obtained for each prefecture was as follows: 656 for Hetian, 449 for Kashi, and 653 for Aksu at the level of 3% dissimilarity. Hetian and Kashi shared 193 OTUs, Hetian and Aksu shared 287, and Kashi and Aksu shared 275. All three sites shared 166 OTUs. Two primary phyla were common to the three areas: Ascomycota and Basidiomycota. Chytridiomycota was present in soil samples from Hetian and Aksu. Glomeromycota was present only in samples from Hetian. Chao and Shannon's indices indicate that the diversity of fungal communities in Hetian was higher than in Kashi and Aksu. We also identified differences between growth ages in the same sampling area. For the soil samples from Hetian prefecture, the dominant phyla were the same for samples He-3 and He-8, except for Chytridiomycota and Glomeromycota, which were identified only in the He-3 sample. However, the dominant phyla had an unbalanced distribution between the 3- and 8-year-old samples.* Ascomycota* was the most abundant phylum in each sample, accounting for 73.2% in Ka-8 and 71.1% in He-3. The proportion of Basidiomycota differed in He-3 (4.32%) and He-8 (20.3%).

### 3.4. Relationships between Fungal Communities and Environmental Variables

**Figure 6:**
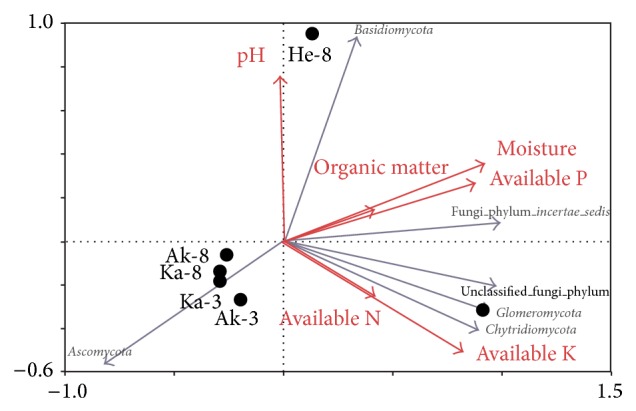
Redundancy analysis (RDA) of abundant fungal phyla and soil properties for individual samples from 3 sampling areas.

Redundancy analysis revealed a significant correlation between soil properties and the abundance of fungal phyla (Figure 6). The abundance of phyla in He-3 correlated with available K and N, and the abundance of phyla in He-8 correlated with pH. The fungal communities in other soil samples correlated negatively with organic matter, moisture, and available P. We found that soil organic matter, moisture, and available P had negative effects on* Ascomycota* abundance and positive effects on the fungi incertae sedis. The abundance of Chytridiomycotaand Glomeromycota correlated with available K and N content. Furthermore, the relative abundance of Basidiomycota correlated with soil pH. Redundancy analysis showed that the sampling sites had less influence on the fungal communities than soil properties, supporting environmental variation as the major determinant of fungal community structure.

## 4. Discussion

The rhizosphere is a critical interface that supports the exchange of resources between plants and their associated soil environment [32]. Rhizosphere microorganisms can greatly affect plant growth by transforming nutrients, contributing to soil organic matter formation, and acting as root pathogens or antagonists of root pathogens. In this study, we used the 454 pyrosequencing technique to investigate rhizospherefungal diversity and community structure at different growth stages of Xinjiang jujube in three major jujube production areas in China.

At present, the internal transcribed spacer (ITS) regions in the 18S, 5.8S, 28S ribosomal RNA gene cluster of fungi have been validated as the best DNA barcode marker for fungal species identification and successfully in metagenomic studies [33]. The primer pair (ITS1F, ITS2) in our study is fungal-specific for the ITS1 region and amplifies a fragment of c.400 bp. The sequencing yielded 40,488 ITS rDNA; each sample averaged 6,748 reads, with only 189–474 OTUs identified in each soil sample. The number of reads and OTUs was lower than previous reports; for example, Buée et al. [34] obtained 166,350 reads and 600–1000 OTUs in each soil sample. In this study, none of the rarefaction curves reached saturation, indicating that further sampling would have revealed additional diversity. Our results are in concordance with previous reports [27, 35, 36]; however, the Shannon index curves became saturated, suggesting that increasing the amount of sequencing reads would not help identify fundamentally new species. We thus concluded that our results are an accurate reflection of the fungal communities they represent.

Sequence analyses revealed that the majority of recovered fungal sequences belonged to the Dikary (Ascomycota, Basidiomycota), which were the most abundant phyla in all samples, accounting for 94.5%. Ascomycota was the most abundant phylum (89.7% of OTUs), whereasBasidiomycota accounted for a much smaller percentage (4.9%). These results were in agreement with those of Schadt et al. [37] who also found a large proportion of Ascomycota in 125 cloned fungal sequences from soils. In contrast, O'Brien et al. [38] used Sanger sequencing and found the proportion of Basidiomycota to be larger than that of Ascomycota. The Chytridiomycota and Glomeromycota were probably underestimated in our data. Chytridiomycotawas found in samples He-3 (0.1%) and Ak-3 (0.03%), while Glomeromycotawas found in He-3 (0.05%) and Ka-8 (0.11%). In addition, there were large proportions of unclassified fungi in He-3 and He-8. A collection of curated sequences for fungal identification is urgently needed, or perhaps the database is inadequate. Because there are fewer fungal phyla, we analyzed the fungal community structure down to the order level. Hypocreales was the most abundant fungal order in contrast to Taylor [39], who reported that Capnodiales was the most abundant fungal order and Hypocrealeswas present in low proportion. The large proportion of unknown fungi found in this and other soil surveys reveals the need for a collection of curated sequences for fungal identification. Pathogenic fungi such as* Aspergillus* and* Schizophyllum *werealso detected. The highest number of sequences (40 reads) matched* Aspergillus, *a known pathogen that causes jujube rot. From the RDA, we know that the function of the fungal populations had links with environmental parameters. For example,* Aspergillus *belongs to Ascomycota which correlated negatively with organic matter, moisture, and available P; therefore we inferred that increasing the soil organic matter, available P, and irrigation can prevent the jujube disease.

Numerous studies have shown that environmental factors shape community structure [40–42]. In our study, an RDA of the environmental variables indicated that pH, available N, available K, available P, organic matter, and moisture were important for the phylogenetic diversity of fungi. A difference in organic matter composition and functioning was reported previously [43]. It is essential to analyze identical numbers of sequences from all samples because diversity estimates always scale up with changes in sampling depth. Our study provides an outline of the fungal diversity and community of the rhizosphere of Xinjiang jujube.

## 5. Conclusions

The aim of this research was to reveal the fungal communities living in the rhizosphere of Xinjiang jujube. 40,488 fungal ITS pyrotag reads were yielded by pyrosequencing. The results revealed the low fungal diversity in this areas. The most abundant fungal phylum was Ascomycota, and the most abundant order was Hypocreales. And the fungal communities were different from other studies. By comparison of fungal communities between different sampling areas, we found that the diversity of fungal communities in Hetian was higher than in Kashi and Aksu. Meanwhile, we discussed the relationship between fungal communities and environmental variables. Furthermore, we hope that our results will provide a reference for future research on the diversity of fungal communities, which thrive in the rhizosphere.

## Figures and Tables

**Table 1:** Diversity and richness index of fungal community from 6 soil samples.

Sites	Location	Sample ID	Cutoff	OTUs	ACE	Chao	Shannon	Coverage
Hetian	37°07′ N 79°56′ E	He-3	0.03	474 ± 14^a^	1064.9 ± 35.2^a^	751.1 ± 35.3^a^	4.37 ± 0.01^a^	0.969
		He-8	0.03	344 ± 33^c^	778.6 ± 60.5^c^	578.0 ± 46.5^b^	3.80 ± 0.03^b^	0.976

Kashi	38°11′ N 77°16′ E	Ka-3	0.03	314 ± 16^c^	788.9 ± 30.3^c^	558.6 ± 36.1^b^	2.98 ± 0.04^c^	0.976
		Ka-8	0.03	189 ± 24^d^	287.4 ± 47.1^d^	291.7 ± 68.8^c^	2.38 ± 0.05^d^	0.988

Aksu	80°11′ N 40°47′ E	Ak-3	0.03	418 ± 28^b^	1040.9 ± 51.8^a^	763.0 ± 72.6^a^	3.71 ± 0.02^b^	0.970
		Ak-8	0.03	399 ± 30^b^	927.4 ± 29.7^b^	740.3 ± 34.9^a^	3.60 ± 0.05^b^	0.972

Statistically significant differences (*P* < 0.05) between 6 different soil samples from 3 areas. Different letters (a, b, c, and d) in column indicate significant difference (*P* < 0.05) between sampling sites according to Duncan's multiple comparison.
